# Transcriptional Analysis of Masson Pine (*Pinus massoniana)* under High CO_2_ Stress

**DOI:** 10.3390/genes10100804

**Published:** 2019-10-13

**Authors:** Fan Wu, Xiaobo Sun, Bingzhang Zou, Peihuang Zhu, Nengqing Lin, Jingquan Lin, Kongshu Ji

**Affiliations:** 1 Key Laboratory of Forestry Genetics & Biotechnology of Ministry of Education, Co-Innovation Center for Sustainable Forestry in Southern China, Nanjing Forestry University, Nanjing 210037, China; eiknarf@126.com (F.W.); sun15336229768@163.com (X.S.); zphzhupeihuang@163.com (P.Z.); 2 Baisha state-owned forest farm, Shanghang 364200, China; zbz808@163.com (B.Z.); lnq196308@126.com (N.L.); LJQ1981722@126.com (J.L.)

**Keywords:** Masson pine, *Pinus massoniana*, CO_2_ stress, transcriptional analysis

## Abstract

To explore the molecular mechanism of the response of Masson pine (*Pinus massoniana*), the main coniferous tree in southern China, to high CO_2_ stress, transcriptome sequencing was carried out to analyze the genome-wide responses of annual seedlings under different durations (0 h, 6 h, 12 h and 24 h) of high CO_2_ stress. The results showed that a total of 3080/1908, 3110/2115 and 2684/1483 genes were up-/down-regulated after 6 h, 12 h and 24 h of treatment, respectively, compared with control check group (CK, 0 h). Kyoto Encyclopedia of Genes and Genomes (KEGG) analysis showed that most of these differentially expressed genes (DEGs) were enriched in energy metabolism, carbohydrate synthesis, cell wall precursor synthesis and hormone regulation pathways. For energy metabolism, the expression of most genes involved in photosynthesis (including the light reaction and Calvin cycle) was generally inhibited, while the expression of genes related glycolysis, the tricarboxylic acid (TCA) cycle and PPP pathway was up-regulated. In addition, the increase in the CO_2_ concentration induced the up-regulation of gene expression in the sucrose synthesis pathway. Among all starch synthesis genes, *GBSS* (granule-bound starch synthase) had the highest expression level. On the other hand, during the synthesis of hemicellulose and pectin (cell wall precursor substances), the expression levels of *GMD* (GDP-mannose 4,6-dehydratase), *MGP* (Mannose-1-phosphate guanylyl transferase) and *RHM* (Rhamnose biosynthetic enzyme) were the highest, suggesting that the synthesis of the raw materials hemicellulose and pectin in Masson pine under stress were mainly supplied by GDP-Man, GDP-Fuc and UDP-Rha. Finally, stress inhibited gene expression in the ABA (Abscisic Acid) synthesis pathway and induced gene expression in the GA (Gibberellin), SA (Salicylic acid), BR(Brassinolide) and MeJA (Methyl Jasmonate) pathways. Stomatal switches were regulated by hormonal interactions. This experiment elaborated on the response and molecular mechanism of Masson pine to CO_2_ stress and aided in screening carbon sequestration genes for the corresponding molecular research of Masson pine in the future.

## 1. Introduction

As humans enter an industrial society, environmental issues have become increasingly prominent. Global warming has been widely monitored by governments and the public worldwide and has risen to become one of the most important political, diplomatic, and economic issues [1]. According to the Global Carbon Project (GCP), global CO_2_ emissions from fossil fuels and industry reached 36.8 Gt in 2017, an increase of approximately 65% from the baseline year (1990) of the Kyoto Protocol [2]. And atmospheric CO_2_ concentrations will rise from 380 ppm today to 550 ppm by 2050 [3]. These numbers are increasing because of the overuse of fossil fuels. Therefore, mitigating global warming caused by the increase in the CO_2_ concentration in the atmosphere has become a serious global challenge.

Studies have shown that elevated CO_2_ concentrations cause changes in plant morphological structures [4], reduce stomatal conductance [5] and the leaf nitrogen metabolism rate [6] and impact other reactions. To maintain normal growth and development, plants have developed a series of physiological, biochemical and molecular regulatory mechanisms, including stomatal regulation, ion homeostasis, signal transduction, etc [7]. Tolerance to high CO_2_ concentrations is a complex trait controlled by genes that play important roles in CO_2_ stress responses in various plants, including heat shock proteins [8], WRKY [9,10], and other photosynthetic-related genes [11]. These previous studies have indicated that the overexpression of these genes could increase the resistance of plants to CO_2_ stress and that the tolerance of plants to high concentrations of CO_2_ could be improved by transgenic and molecular marker-assisted breeding.

Masson pine (*Pinus massoniana*) is a conifer species distributed in 17 provinces, autonomous regions and municipalities located south of the Qinling Mountains in China. It thrives in a warm and humid climate, growing in arid, barren gravel soil and sandy soil. It is a pioneer species for restoring forests in barren hills [12]. Studies have shown that the carbon sequestration capacity of Masson pine is much higher than the average carbon sequestration of forests in China [13]. The carbon sequestration of each organ of Masson pine is between 533.93 and 568.08 g·kg^−1^, which is higher than the carbon content of 32 common tree species (444.0~494.5 g·kg^−1^) [14]. In recent years, there have been many studies on the physiological and biochemical responses to CO_2_ stress and the screening of candidate CO_2_-resistance genes [15,16,17]; however, few studies on the CO_2_ tolerance mechanism and transcriptome response of Masson pine have been reported. In this study, a next-generation transcriptome sequencing analysis of Masson pine under high CO_2_ stress was evaluated using the Illumina HiSeq sequencing platform. The transcriptome results were used to identify genes that might be involved in the response to CO_2_ and to clarify the possible molecular mechanisms involved in the adaptation to CO_2_ stress. To verify the accuracy of the sequencing results, we selected several genes for quantitative real-time PCR (qRT-PCR) verification. The results improve our understanding of environmental acclimation mechanisms in Masson pine and serve as a molecular-level reference to inform future work on the enhancement of CO_2_ tolerance in Masson pine.

## 2. Materials and Methods 

### 2.1. Plant Material and Experimental Conditions

One-year-old Masson pine seedlings, obtained from the seed orchard of Baisha state-owned forest farm, Shanghang, Fujian Province, China (25°15′ N, 116°62′ E), were used in this study. Individuals of the same clones with similar heights, uniform growth and strong growth potential were selected as the test materials and subsequently moved into a growth chamber to recover for 15d. The growth conditions were 10 h light/14 h dark cycles at 25 °C in the chamber. Air containing about two times of the chamber CO_2_ concentration before experiment was aerated into the growth chamber constantly for at least 24 h. The CO_2_ concentration in the chamber was monitored by an infrared CO_2_ analysis reader (SenseAir, Delsbo, Sweden). The seedlings were sampled after 6 h, 12 h and 24 h of treatment with the high CO_2_ concentration, and the needles were selected for downstream experiments.

### 2.2. Total RNA Isolation, Complementary DNA Library Preparation and Sequencing

Total RNA from four treatments (0 h, 6 h, 12 h and 24 h) seedlings (Among them, 0h treatment was considered as the control check group (CK group)) with three biological replicates for each treatment was extracted using the Plant RNA Isolation Kit (Tiangen, Beijing, China). Sequencing library were constructed using RNA Library Prep Kit for Illumina (NEB, Boston, MA, USA). Then the libraries were sequenced using a Hiseq 4000 (Illumina, San Diego, CA, USA), and generated 150 bp paired-end reads. To get clean reads, sequences with length less than 30 bp, reads with *N* ratio over 10% and without inserted fragments due to reasons such as connector self-connection and adapter sequences were removed using SeqPrep (https://github.com/jstjohn/SeqPrep) and Sickle (https://github.com/najoshi/sickle) [18]

### 2.3. Transcriptome Assembly and Functional Annotation

De novo assembly of all the clean reads was conducted using Trinity version 2.5.0 (https://github.com/trinityrnaseq/trinityrnaseq/wiki) based on all parameters set as their defaults [19]. Transcripts corresponding to paralogous genes were sorted out to finally obtain the assembly sequences. TransRate software version 1.0.3 (http://hibberdlab.com/transrate/) was used to filter and optimize the initial assembly sequences obtained from Trinity [20], and the assembled sequence was evaluated using BUSCO (Benchmarking Universal Single-Copy Orthologs) version 3.0.2 (http://busco.ezlab.org) [21]. Both of the two softwares ran with their default parameters. 

To functionally annotate genes, the sequences were BLASTed in Kyoto Encyclopedia of Genes and Genomes (KEGG) (https://www.genome.jp/kegg/) public databases. KEGG enrichment analysis for the DEGs was carried out by KOBAS version 3.0. In this software, False-positive were assessed by BH (FDR correction with Benjamini/Hochberg) method with a cut-off *E*-value of 10^−6^. When the *p*-adjust (the adjusted *p*-value) of a KEGG pathway was less than 0.05, we considered this KEGG pathway was significantly enriched.

### 2.4. Differential Expression Analysis

The gene expression level for each sample was determined according to the transcripts per million reads (TPM) using RSEM version 1.2.31 (Univ Wisconsin, Madison, WI, USA) with all parameters set as its defaults [22], in which the read counts were normalized using the edgeR package with the Trimmed Mean of M-values method, and then the length of the gene (L) and the normalized counts (N) were used to calculate the TPM (TPM = 10^6^ × (N_i_/L_i_)/∑(N_j_/L_j_)). The DEGseq *R* package version 1.10.1 was used to analyze differential expression of different samples [23]. The significant differential expression threshold was set as *q*-value < 0.005 and |log_2_(foldchange)| ≥ 1 [24]. The heatmap and differential expression of genes among samples were analyzed using “pheatmap” *R* package version 1.0.12 (Massachusetts General Hospital, Boston, MA, USA). 

### 2.5. Quantitative Real-Time PCR Validation

To validate the RNA-sequencing (RNA-seq) results, the expression levels of nine genes were measured using qRT-PCR. Reaction mixtures consisted of 10 μL of 2× TOP Green qRT-PCR SuperMix (TOYOBO Biotech, Shanghai, China), 0.4 μL of forward primer and reverse primer, 2 μL of complementary DNA (cDNA), 0.4 μL of 50× Passive Reference Dye and 6.8 μL of ddH_2_O. The PCR program was set up in six stages: (1) 94 °C for 30 s (preincubation), (2) 94 °C for 5 s, (3) 55 °C for 15 s, (4) 72 °C for 10 s, repeated 40 times (amplification), (5) 95 °C for 0.5 s, and (6) 60 °C for 1 min (melt). The PCR quality was estimated based on melting curves. *TUA* (Alpha-tubulin) was used as the internal control [25]. The gene-specific primers employed are shown in Table A1 from Appendix A. Three independent biological replicates and three technical replicates for each biological replicate were run. Quantification was achieved using comparative cycle threshold (Ct) values, and gene expression levels were calculated using the 2^−∆∆Ct^ method [25]. The significance was determined by *t*-test using SPSS statistical software (IBM, New York, NY, USA) (*p* < 0.05).

## 3. Results

### 3.1. Transcriptome Sequencing and De Novo Assembly

The cDNA libraries of the four treatments (0 h (CK), 6 h, 12, and 24 h) were sequenced and generated a total number of 49,314,299, 47,459,322, 45,980,036, and 60,876,932 raw reads and 48,795,571, 46,976,134, 45,496,760, and 60,205,674 clean reads, respectively (Table 1). The raw data and sequences can be found online at the NCBI (https://www.ncbi.nlm.nih.gov/) Sequence Read Archive (SRA) database (accession number PRJNA561037). Compared with the reference sequences obtained from Trinity assembly, there were generated a total number of 16,879,027, 16,459,249, 15,859,340 and 21,108,414 mapped reads of 0 h, 6 h, 12 h, and 24 h treatments, respectively. An average mapped ratio of 70% was obtained. The Q30, a key parameter that represents the quality of sequenced bases, was 94.04%, 94.22%, 93.86% and 93.4% for the 0 h, 6 h, 12 h, and 24 h treatments, respectively (Table 1). 

The Trinity software generated 140,863 transcripts with an average length of 891 bp and an N50 of 1463 bp. In total, 92,424 unigenes were obtained in the range of 201~15,491 bp. Of these, 48,592 (52.57%) were less than 500 bp, 22,267 (24.09%) were 501~1000 bp, 12,887 (13.94%) were 1001~2000 bp and the remaining 8678 (9.39%) were >2000 bp (Table 2). TransRate and software BUSCO evaluated the assembly results, and transcript score was 0.20045 and 77.7%, respectively, and unigenes was 0.30498 and 74.2%, respectively (Table 2). According to previous reports [26,27], in Masson pine, unigene obtained by transcriptome sequencing under other stress treatments ranged from 70,896 to 101,806. Our results were similar to them. Combined with the N50 and Q30, we believe that the sequencing results are relatively reliable and could be further analyzed.

### 3.2. Gene Expression and KEGG Enrichment Analysis under CO_2_ Stress

**Figure 1:**
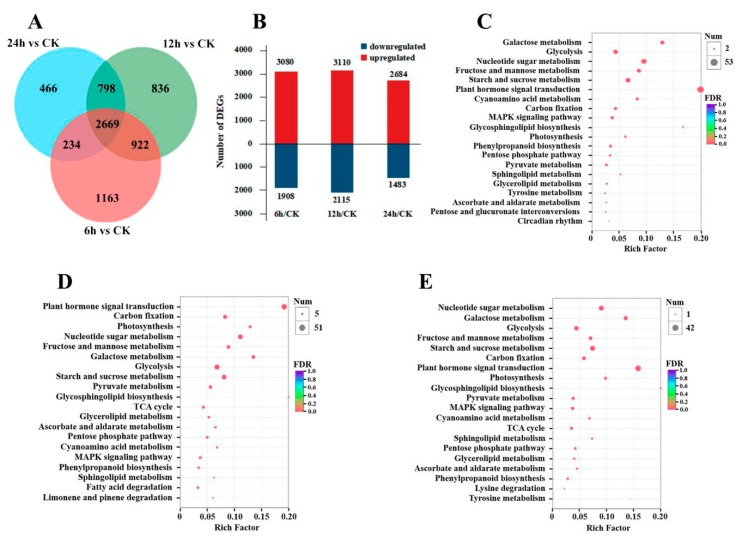
Differential gene expression in seedlings under high CO_2_ stress. (**A**) Venn diagram of differentially expressed genes. (**B**) Statistical map of differentially expressed genes between different comparisons. Red and blue represent up- and down-regulated expression, respectively. (**C**–**E**) The top 20 pathways in the KEGG enrichment analysis of CK compared with the 6 h, 12 h and 24 h treatments, respectively. CK: 0 h or control check group.

A total of 7088 genes were differentially expressed between the samples from the three CO_2_ stress treatments and the control samples. Of these, 4988, 5225 and 4167 genes were differentially expressed between 6 h and CK, 12 h and CK, and 24 h and CK treatments, respectively (Figure 1A). Among the differentially expressed genes (DEGs), 3080/1908, 3110/2115 and 2684/1483 genes were up-/down-regulated at 6 h, 12 h, and 24 h, respectively, compared with CK (Figure 1B). Gene enrichment analysis of the DEGs based on KEGG analysis revealed that these genes were mainly involved in several pathways at different time points, including photosynthesis, carbon fixation (the Calvin cycle), glycolysis, the tricarboxylic acid (TCA) cycle, starch and sucrose metabolism, fructose and mannose metabolism, galactose metabolism and plant hormone signal transduction, etc. (Figure 1C,D,E). In the three KEGG enrichment Figures that compare the different treatment time points with CK, all of the above pathways ranked within the top 20 pathways (except the “TCA cycle” in 6 h/CK, Figure 1C), indicating that energy metabolism, carbohydrate synthesis, cell wall synthesis and hormone regulation may be the main metabolic activities in Masson pine under high CO_2_ stress.

### 3.3. Energy Metabolism under Elevated CO_2_ Stress

**Figure 2:**
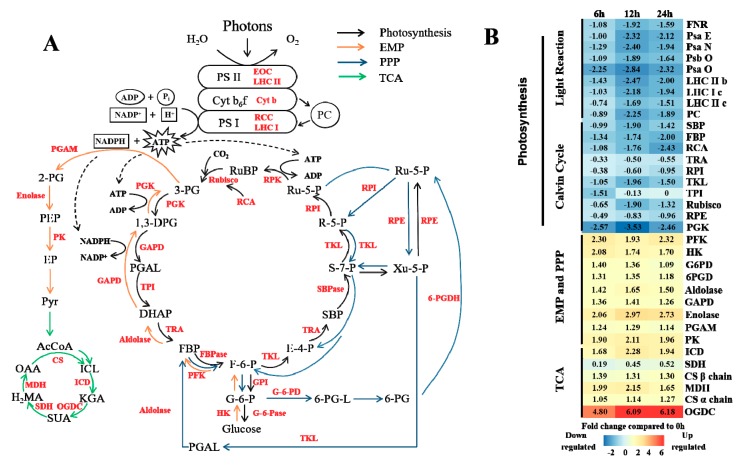
Influence of high CO_2_ concentration on energy metabolism. (**A**) The main energy pathways in plants. Black, orange, blue and green arrows represent photosynthesis, the Embden-Meyerhof-Parnas (EMP) pathway, the pentose phosphate (PPP) pathway and the tricarboxylic acid (TCA) cycle, respectively. The different enzymes are shown in red font. (**B**) Expression changes in the genes involved in metabolic pathways in response to stress. White indicates no change, red up-regulation, and blue down-regulation in each treatment, as shown in the color bar for a log_2_ fold change scale. The abbreviations in the figure are shown in Table A2 from Appendix A.

Figure 2 shows the energy metabolic pathways and the general pattern of the relative changes in the related gene expression patterns in Masson pine under high CO_2_ concentration conditions. Notably, in the transcriptome data, the pathways involved in energy metabolism were polarized. After Masson pine had been exposed to CO_2_ stress, in the photosynthesis pathways, including the photoreactions and Calvin cycle (Figure 2A, black arrow), the relative expression of each gene in the metabolic pathway except for *GAPD* (glyceraldehyde-3-phosphate dehydrogenase) showed a downward trend (Figure 2B). GAPD catalyzes 3-phosphoglyceraldehyde (PGAL) to 1,3-diphosphoglycerate (1,3-DPG) in the Calvin cycle and 1,3-DPG to dihydroxy-acetone phosphate (DHAP) in the Embden-Meyerhof-Parnas (EMP) pathway (Figure 2A, orange arrow). Since *GAPD* plays a role in these two metabolic pathways, the change in its expression patterns may be the result of superposition. Moreover, the gene expression patterns involved in both photosynthesis and other metabolic pathways (such as *RPI* (Ribulose Phosphate Isomerase) or *RPE* (Ribulose Phosphate Epimerase)) decreased more slowly than those involved only in photosynthesis (such as *RCA* (Rubisco Activase) or *FBP* (Fructose-1,6-diphosphate)). On the other hand, the relative expression levels of genes in other pathways involved in energy anabolism, including the EMP pathway, the pentose phosphate (PPP) pathway (Figure 2A, blue arrow) and TCA (Figure 2A, green arrow), generally increased (Figure 2B). Among them, the expression rate of *OGDC* (α-ketoglutarate dehydrogenase) increased the fastest and was 4.8, 6.09 and 6.18 times that of CK at 6 h, 12 h and 24 h, respectively. In general, the omics data revealed that energy metabolism was strongly enhanced for contributing to elevated CO_2_ tolerance in Masson pine, except for the light reaction and Calvin cycle.

### 3.4. Biosynthesis of Sucrose, Starch and Cell Wall Components under Elevated CO_2_ Stress

**Figure 3:**
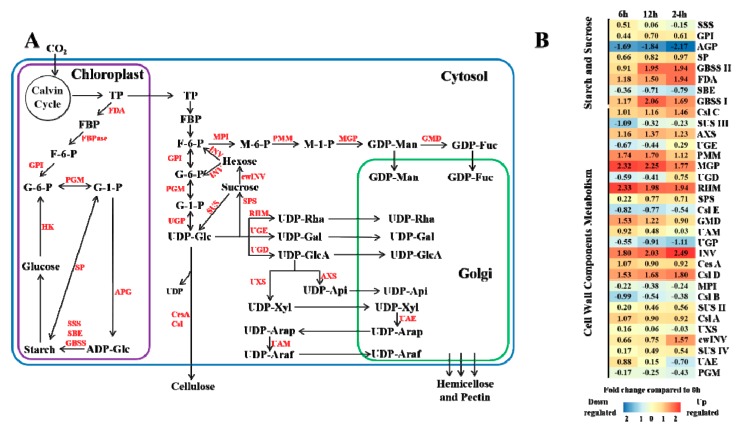
Influence of high CO_2_ concentration on sucrose, starch and cell wall components. (**A**) The sucrose, starch and cell wall component biosynthesis pathways according to Evžen [28] and Jana [29]. The blue, purple and green rectangles represent the cytosol, chloroplast and Golgi, respectively. The different enzymes are shown in red font. (**B**) Expression changes in the genes involved in metabolic pathways in response to stress. White indicates no change, red up-regulation, and blue down-regulation in each treatment, as shown in the color bar for a log_2_ fold change scale. The abbreviations in the figure are shown in Table A2 from Appendix A.

The expression levels of the genes in the sucrose and starch synthesis pathways generally showed a trend of up- or slightly down-regulation under CO_2_ stress conditions compared with CK, except for *AGP* (Adenosine Diphosphoglucose Pyrophosphorylase) and *SBE* (Starch Branching Enzyme) (Figure 3B). AGP catalyzes adenosine diphosphate glucose (ADP-Glc) from glucose-1-phosphate (G-1-P), and its expression continued to decline with increasing stress time. On the other hand, among the genes in starch metabolic pathway, the expression of *GBSS* (Granule-bound Starch Synthase) was higher than *SSS* (Soluble Starch Synthase) and *SBE*.

After a series of reactions, triose phosphate was transformed into UDP-Glc in the cytoplasm (Figure 3A), and then it was catalyzed to sucrose by sucrose-phosphate synthase (SPS). After being subjected to stress treatment, the expression levels of *SPS* at 6 h, 12 h and 24 h were no significant difference (Figure 3B). Meanwhile, sucrose can be transformed to UDP-Glc and hexose by sucrose synthase (SUS) and cell wall invertase (cwINV), and hexose can then be catalyzed by cytosolic invertase (INV) to form fructose-6-phosphate (F-6-P) and glucose-6-phosphate (G-6-P), which are the synthetic precursors of UDP-Glc (Figure 3A). In the above series of reactions, the expression of *SUS*, *cwINV* and *INV* were up-regulated in different degrees under CO_2_ stress compared with CK. In addition, UDP-Glc is the precursor for cellulose synthesis, which is catalyzed by cellulose synthase complex (CSC), including cellulose synthase subunit (CesA), cellulose synthase (Csl) and its isoenzymes (Figure 3A). The experimental results showed that the expression levels of *CesA*, *Csl A*, *Csl C* and *Csl D* were up-regulated with increasing stress time. However, *Csl B* and *Csl E* decreased at the same time (Figure 3B). In general, the omics data revealed that gene expression patterns in sugar and cell wall component metabolic pathways were enhanced under elevated CO_2_ stress in Masson pine.

### 3.5. Hormone Regulation under Elevated CO_2_ Stress

**Figure 4:**
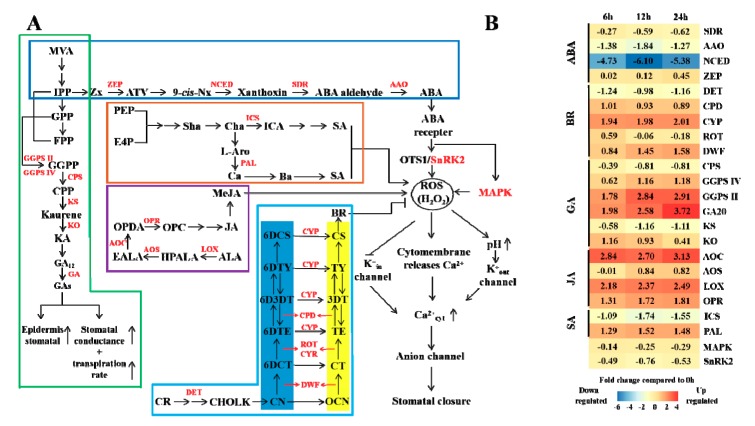
Influence of high CO_2_ concentration on hormone and stomatal regulation. (**A**) The hormone biosynthesis pathways and stomatal regulation mechanism according to Zhao [35]. The mazarine, green, brown, purple and wathet frame represents ABA, GA, SA, JA and BR synthetic pathways, respectively. The blue and yellow block represents advanced and early C-6 oxidation pathway in BR biosynthesis, respectively. The different enzymes are shown in red font. Sharp and *T*-shaped arrows indicate positive and negative regulation, respectively. (**B**) Expression changes in the genes involved in metabolic pathways in response to stress. White indicates no change, red up-regulation, and blue down-regulation in each treatment, as shown in the color bar for a log_2_ fold change scale. The abbreviations in the figure are shown in Table A2 from Appendix A.

Based on previous studies [30,31,32,33], combined with KEGG analysis results, the expression patterns of 5 plant hormones (ABA, GA, SA, BR and JA) and their corresponding synthesis genes under CO_2_ stress were analyzed in this study. In the ABA synthesis pathway, except for the expression of *ZEP* (zeaxanthin epoxidase) that was slightly up-regulated (no significant difference from CK), all other genes were down-regulated. Among them, *NCED* (9-cis-epoxycarotenoid dioxygenase) decreased most obviously (Figure 4B). and the log2 fold changes in expression compared to CK decreased 4.73, 6.10 and 5.38 times at 6 h, 12 h and 24 h, respectively. 

On the other hand, the genes expression pattern of the other four hormones showed an increasing trend under CO_2_ stress. The key enzyme in the GA metabolic pathway is geranyl geranyl pyrophosphate (GGPS), which catalyzes the synthesis of geranylgeranyl pyrophosphate (GGPP) from isopentenyl pyrophosphate (IPP), geranyl pyrophosphate (GPP) and farnesyl pyrophosphate (FPP). Under CO_2_ stress, *GGPS* expression increased continuously with time (Figure 4B). 

As shown in Figure 4A, SA can be synthesized via two routes, by isochorismic acid (ICA) and by cinnamic acid (Ca), benzoic acid (Ba), etc. The specific genes involved in the first pathway have not yet been thoroughly studied [34], while the second pathway has been well researched. Under CO_2_ stress, *PAL* expression continued to increase, reaching a peak at 12 h and then stabilizing (there was no significant difference between 24 h and 12 h). Due to the expression pattern of *PAL*, we speculated that the pathway used for SA synthesis in Masson pine under CO_2_ stress was mainly the second pathway.

The BR synthesis pathways include early (Figure 4A, yellow block) and advanced (blue block) C-6 synthetic pathways [35]. Intermediate metabolites in the advanced pathway could be converted to corresponding metabolites in the early pathway. However, at the upstream pathway, the conversion efficiency was not very high because the expression levels of *ROT* (C-23 hydroxylase) was down-regulated with increasing treatment time. In contrast, downstream of the pathway, due to the increase in *CYP* expression (Figure 4B), BR might be synthesized through the early pathway. Overall, both metabolic pathways showed increased efficiency under CO_2_ stress (Figure 4B).

All genes in the JA (MeJA) synthetic pathway were up-regulated under CO_2_ stress. Among them, *AOC* (allene oxide synthase) was the most significantly up-regulated. At 6 h, the relative expression level of *AOC* was 2.84 times that of CK and then increased, and may continue to increase with increased treatment time. Within 24 h, the expression of *AOC* was far higher than that of the other genes in the pathway (Figure 4B). It could be speculated that *AOC* might be a key gene involved in the induction of JA production in Masson pine under high CO_2_ concentrations.

### 3.6. Validation by Quantitative Real-Time PCR

**Figure 5:**
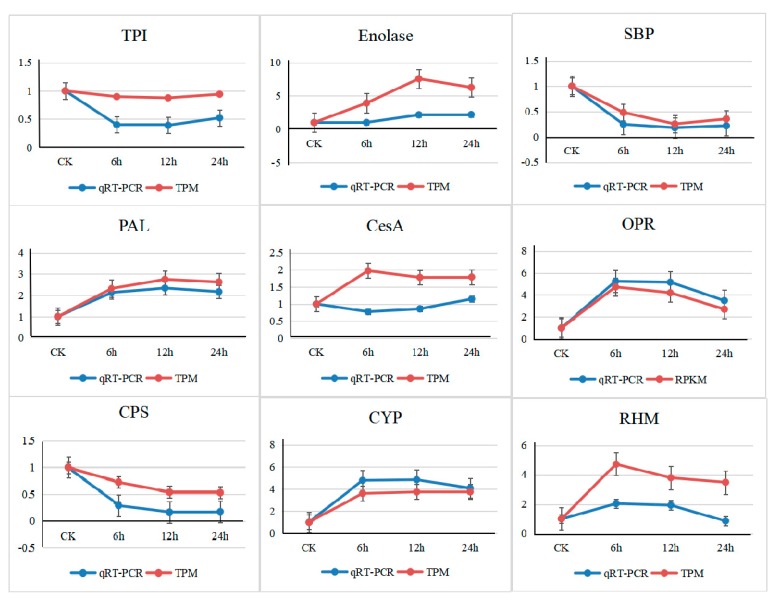
The expression changes in the 9 randomly selected genes were determined using quantitative real-time PCR (qRT-PCR) results and sequencing data. The x-axis represents different processing times, and the y-axis represents changes in gene expression under CO_2_ stress. The data show the fold change in the expression of each gene under high CO_2_ relative to control conditions. Error bars represent standard deviations. Red indicates the RNA-sequencing results under the TPM (transcripts per million reads) algorithm, and blue indicates the qRT-PCR results.

To verify the reliability of the transcriptome data, nine genes showing significant up- or down-regulation in the stressed seedlings were randomly chosen for qRT-PCR analysis (Figure 5). Among them, *PAL* showed constitutively up-regulated expression, and *CYP* increased at first and reached maximum expression at 6 h, after which there was no significant change. Compared with CK, 6 h, 12 h and 24 h CO_2_ treatments had up-regulated levels of *RHM* and *OPR*, and the relative expression levels of these genes were higher at 6 h than at 12 h and 24 h. On the other hand, enolase had the same trend as *RHM* and *OPR*, except that the maximum value appeared at 12 h. *CPS* and *SBP* showed constitutively down-regulated expression, and *TPI* (triose-phosphate isomerase) showed a trend of first decline and then increase. Among all genes, the qRT-PCR results of 6 genes (*CYP*, *CPS*, *OPR*, *PAL*, *SBP* and *TPI*) were very close to the RNA-seq (via the TPM algorithm) results. The expression trend of *RHM* and enolase was similar to that of RNA-seq, but the fold change in qRT-PCR expression was lower than that in the RNA-seq data. The qRT-PCR results of *CesA* were quite different compared with the RNA-seq results. In general, the RNA-seq data were similar to the gene expression trend shown by qRT-PCR analysis, indicating that the results of RNA-seq analysis were effective.

## 4. Discussion

### 4.1. Effects of Elevated CO_2_ on Masson Pine Energy Metabolism

The results of this study showed that in the major energy metabolism pathways of Masson pine, the expression levels of most genes involved in photosynthesis, including light and dark reactions, showed a significant downward trend under CO_2_ stress. We speculate the main cause of this phenomenon should be the decrease in the expression of Rubisco, which is a key enzyme in photosynthetic carbon assimilation, directly affecting the photosynthetic efficiency of Masson pine [36]. Combined with the up-regulated of key genes in the carbohydrate metabolism during the same period (Figure 3B), it leads to the activation of its signaling pathway, which inhibits the expression of *RbcS* (Rubisco small subunit) and other photosynthesis-related protein-coding genes through hexokinase transmission, thereby affecting the photosynthetic efficiency [37]. 

Significantly, previous studies [38,39] have shown the expression of *Rubisco* will show an up-regulated trend under the high CO_2_ treatment condition. But the results of this experiment are the opposite. This may be because the expression of carbon sequestration gene is greatly affected by the leaf age [40]. The photosynthetic acclimation of coniferous leaves in one-year needles under high CO_2_ treatment was more obvious than that in mature coniferous leaves [41]. According to the experimental results, the expression of each gene in photosynthesis showed a downward trend when the sample was analyzed at the shortest detection time (6 h). Therefore, we speculate the photosynthetic acclimation should be completed within 6 h.

However, in other energy metabolism pathways of Masson pine, the expression levels of various genes under high CO_2_ stress generally showed an upward trend. Fukayama et al. [42] obtained similar results in an experiment on the effect of CO_2_ on rice, but the difference in gene expression was inferior to our study, possibly because rice belongs to herbage, and the adaptability of rice to CO_2_ stress is higher than that of Masson pine. Previous studies found that a high concentration of CO_2_ promoted the production of a large amount of sucrose in leaves, and the increase in sucrose concentration induces the expansion of glycolytic flux in plants and simultaneously increases the distribution of carbon in the organic acid and TCA cycles, thereby regulating the increase in related gene expression [43]. 

### 4.2. Effects of Elevated CO_2_ on Masson Pine Carbohydrate and Cell Wall Component Synthesis

In photosynthetic cells, fixed carbon eventually transforms into sucrose and starch. These compounds are the main form of carbohydrate transport and storage in advanced plants. *SPS*, *SUS* and *INV* are considered to be the key genes in the sucrose synthesis pathway [44]. The results of our experiment suggest that there were no significant differences in *SPS* and *SUS* levels between the CK and different treatments, while *INV* showed an increased difference. This result suggested that, sucrose produced by photosynthesis was more likely to be decomposed into glucose-6-phosphate (G-6-P) and fructose-6-phosphate (F-6-P), and then participated in subsequent reactions is the main metabolic direction of sucrose under high CO_2_ stress. Van et al. [45] found that INV was significantly up-regulated and SUS was relatively inhibited in mature leaves of in vitro tomato plantlets with high CO_2_ stress under the condition of providing exogenous sucrose (3%). In addition, other studies also showed that the expression of INV and the contents of glucose and fructose in leaves increased under cold, NaCl, PEG and other stress conditions, which showed similar phenomena to CO_2_ stress conditions [46,47].

In the starch synthesis pathway, three different starch-formed synthetases (GBSS, SSS and SBE) are the key regulatory enzymes. In this study, the expression level of *GBSS* were significantly higher than that of *SBE* and *SSS* under high CO_2_ stress. *GBSS* generates a branchless linear glucan starch chain (amylose) by specific binding to starch granules, which is necessary for the production of amylose [48]. Due to the high expression of *GBSS*, we suspected that, amylose was more inclined to synthesize under high CO_2_ stress in Masson pine. The similar results were confirmed in Cui [49] study. He found that the CO_2_ stress could significantly improve the activity of GBSS in winter wheat, resulting in a large amount of amylose production, and the expression level increased first and then decreased, which was similar to this experiment.

### 4.3. Effects of Elevated CO_2_ on Masson Pine Cell Wall Component Synthesis

The plant cell wall is mainly composed of polysaccharides, which are the largest storage place for photosynthetic carbon fixation [50]. The synthesis of cell wall components is a highly complex process involving multiple enzymes and metabolic intermediates [29]. The major components of the cell wall, such as cellulose, hemicellulose, and pectin, require a variety of ribose compounds, most of which rely on UDP-Glc derivatization (Figure 3A) [29,51]. For the genes regulating UDP-Glc to produce hemicellulose and pectin precursor derivatives, the expression levels of *GMD* and *RHM* were higher than those of others. Due to the increased expression of *RHM* and *GMD* under CO_2_ stress, we speculate that their corresponding products (UDP-Rha and GDP-Fuc) were the main components of cell wall precursors at this stage. However, there is still no unified conclusion on its specific process of influence. Sufficient long-term research and evidence to fully and thoroughly understand the mechanism are required.

### 4.4. Effects of Elevated CO_2_ on Masson Pine Hormones and Stomatal Regulation

In this study, the results showed that except *ZEP*, the expression of each gene in the ABA synthesis pathway tended to decrease with prolonged CO_2_ treatment time, especially *NCED*. The catalysis of 9-*cis*-neoxanthin to Xanthoxin by NCED is a key step in the ABA metabolic pathway in plastids [52].Therefore, a decrease in the expression of *NCED* seriously affects the expression of other genes in the ABA metabolic pathway, which in turn affects ABA accumulation in the cell [53]. Previous studies have shown that ABA is greatly affected by BR when regulating stomatal opening and guard cell physiological states [54]. Elevated CO_2_ led to a sharp increase in the expression of key genes in the BR metabolic pathway, such as *CYP*, which would inhibite ABA from binding to ABI (a kind of serine/threonine protein phosphatase), thereby weakening the effect and signal transmission of ABA [55].

For other hormones, the key genes in their synthetic pathway increased to varying degrees at the same stage. Among them, the expression of *GGPS*, the key gene of GA synthesis, continued to up-regulate with prolonged stress. Studies have shown that GA can divide plant hypocotyl epidermal cells, promote stomatal formation, regulate stomatal density [56], so as to maintain stomatal conductance and the transpiration rate under the condition of elevated CO_2_ concentration. On the other hand, according to our result, the key gene in the JA and SA synthetic pathway, including *AOC*, *LOX* and *PAL*, remained at a high expression level with prolonged stress. It indicated that the reaction proceeded in the direction of JA and SA synthesis, which would cause stomatal closure [57,58,59].

In general, although previous studies have shown that ABA is the most important hormone to control stomatal switching in plants, in Masson pine under CO_2_ stress, the expression level of each gene in the ABA synthesis pathway was basically inhibited. However, this did not affect stomatal closure in Masson pine because the genes regulated other hormones that promote stomatal formation or induce its closure were general up-regulated in the same environment.

## 5. Conclusions

The effect of rising CO_2_ concentrations on plants is known, however, little research has been done on Masson pine. In this study, we tried to explore the molecular response of Masson pine under high CO_2_ stress. The results showed that, the genes expression would generally decrease in photosynthesis pathway (light reaction and Calvin cycle), and generally increase in other energy metabolic pathways, including TCA, EMP and PPP. At the same time, Increased CO_2_ concentration could also promote the gene expression in cell wall precursor synthesis pathway. In addition, CO_2_ stress inhibited the genes expression in the ABA synthesis pathway, but increased in other hormones synthesis pathway (including BR, GA, SA and MeJA), which might regulate stomatal density and stomatal closure. As the first report on the high-throughput sequencing of CO_2_ tolerant Masson pine, this study should provide novel insights into CO_2_ tolerance genes and be a valuable molecular basis for study in Masson pine.

## Figures and Tables

**Table 1:** Summary of sequencing data quality control.

	CK	6 h	12 h	24 h
Raw reads	49,314,299	47,459,322	45,980,036	60,876,932
Raw bases	7,446,459,199	7,166,357,723	6,942,985,436	9,192,416,732
Clean reads	48,795,571	46,976,134	45,496,760	60,205,674
Clean bases	7,292,383,178	7,017,302,463	6,802,876,221	9,000,454,730
Error rate (%)	0.02	0.02	0.03	0.03
Mapped reads	16,879,027	16,459,249	15,859,340	21,108,414
Mapped ratio (%)	0.69	0.70	0.70	0.70
GC content (%)	47.47	46.74	46.24	46.32
Q20 (%)	98.12	98.20	98.06	97.86
Q30 (%)	94.04	94.22	93.86	93.40

**Table 2:** Length distribution and software evaluation of unigenes and transcripts.

Type	Transcript	Unigenes
<500 bp	61,696	48,592
501~1000 bp	37,586	22,267
1001~2000 bp	25,994	12,887
>2000 bp	15,587	8,678
Total	140,863	92,424
Min length (bp)	201	201
Max length (bp)	15,491	15,491
Mean length (bp)	891	935
N50 (bp)	1463	1550
TransRate score	0.20045	0.30498
BUSCO score	77.7%	74.2%

## Appendix A

**Table A1:** Primers used in this study.

Primer		Sequence (5′→3′)
RHM	Forward	TACGAATAGTCTCTGGCTTGTGAG
	Reverse	TCTGGTTGTGTCCTTGACCTAATA
CYP	Forward	TCTATGGTGATCACTGGAGAAAGA
	Reverse	GATGAGAGAATGGTTGAGAATGTG
CPS	Forward	TACTCGGTGTTATAAGTGCAGCTC
	Reverse	CATGTAGCCCTTGACACAAAATAG
OPR	Forward	TACGATACGGGAACAACTACTGAA
	Reverse	TCGAGCTCTAAAAACTGAGGAGAT
PAL	Forward	GAAGCCTGAGTTTACAGATCCATT
	Reverse	CGTAAACCACTTCAATCACTTCAC
CesA	Forward	GGAAGGCTGTACTTTATCCTTCAA
	Reverse	ATGCAAGACCAGATACAAGAGACA
SBP	Forward	TACCAGCCCAATAACAATAACCAC
	Reverse	CTCTCATCCACGAAGCTAATAACC
TPI	Forward	CCCTCTGCCACTTTCTTTATGTC
	Reverse	TCTAAGACTTCCTCACTTCTCCG
Enolase	Forward	AAGAGCTGCAAGGTAAAGTCTGTT
	Reverse	TCTGATTCACCTTCAGCAGTAAAG
qRT-PCR-RHM	Forward	CCACATCCTCACAGTAGAGATAGC
	Reverse	CCGGTGATTACTACCAGAGGTAAC
qRT-PCR-CYP	Forward	AGGCCCTTCCTCAGAGGTTATCT
	Reverse	CCGGAGTTGGTACTAGTCTTGGTA
qRT-PCR-CPS	Forward	GTCTAGAGCGGTTCACTCAGAT
	Reverse	CCTCTCTCCAACTATCACTGTGTC
qRT-PCR-OPR	Forward	GGTACCGTTCTTACTGGTTTGAGG
	Reverse	GATCCTGTAGTTGGCTACACAGAC
qRT-PCR-PAL	Forward	CCCTCAGGTGGAGATTATCAG
	Reverse	CCTCCATGTAGAGCTTTGTCTC
qRT-PCR-CesA	Forward	CCTGTACGGAGTAAGTTTGGTG
	Reverse	ACCAGTGGAGGTAGATATGCTG
qRT-PCR-SBP	Forward	CTACAGAGATAGGAGAGGGGAAAC
	Reverse	CTCCTGTGTATCGGAGTGTGTACT
qRT-PCR-TPI	Forward	CCTCCCACTTCTACTAGGGTTT
	Reverse	ACCAGCCAGGAGTAGTTAAGAGTG
qRT-PCR-Enolase	Forward	GGCCAGACAGATTATAGACAGC
	Reverse	CTCTCATCTCTAGGGCCTCATA

**Table A2:** Abbreviation in this study.

Abbr.	Full Name	Abbr.	Full Name
PS	Photosystem	LEC	Light Harvesting Complex
PC	Plastocyanin	RuBP	Ribulose-1,5-Bisphosphate
3-PG	3-Phosphoglycerate	1,3-DPG	1,3-Diphosphoglycerate
PGAL	3-Phosphoglyceraldehyde	DHAP	Dihydroxy-Acetone Phosphate
FBP	Fructose-1,6-Diphosphate	F-6-P	Fructose-6-Phosphate
E-4-P	Erythrose 4-Phosphate	SBP	Sedoheptulose-1,7-Diphosphate
S-7-P	Sedoheptulose-7-Phosphate	R-5-P	Ribose-5-Phosphate
Ru-5-P	Ribulose-1,5-Phosphate	Xu-5-P	Xylulose-5-Phosphate
6-PG	6-Phosphogluconate	6-PG-L	Gluconolactone-6-Phosphate
G-6-P	Glucose-6-Phosphate	2-PG	2-Phosphoglycerate
PEP	Phosphoenolpyruvic acid	EP	Enolpyruvic Acid
Pyr	Pyruvic Acid	AcCoA	Acetyl CoA
ICL	Isocitric Acid	KGA	α-Ketoglutaric Acid
SUA	Succinate Acid	H2MA	Malic Acid
OAA	Oxaloacetic Acid	RCA	Rubisco Activase
PGK	Phosphoglycerate Kinase	GAPD	Glyceraldehyde-3-Phosphate Dehydrogenase
TPI	Triose-Phosphate Isomerase	TRA	Transaldolase
FBPase	Fructose-1,6-Bisphosphatase	TKL	Transketolase
RPI	Ribulose Phosphate Isomerase	SBPase	Sedoheptulose-1,7-Bisphosphatase
RPK	Phosphoribulokinase	RPE	Ribulose Phosphate Epimerase
6-PGDH	6-Phosphogluconate Dehydrogenase	G-6-PD	Glucose-6-Phosphate-Dehydrogenase
G-6-Pase	Glucose-6-Phosphatase	HK	Hexokinase
PK	Pyruvate Kinase	PGAM	Phosphoglycerate Mutase
CS	Citrate Synthase	ICD	Isocitrate Dehydrogenase
OGDC	α-Ketoglutarate Dehydrogenase	SDH	Succinate Dehydrogenase
MDH	Malate Dehydrogenase	TP	Triose Phosphate
ADP-Glc	Adenosine Diphosphate Glucose	UDP-Glc	Uridine Diphosphate Glucose
M-6-P	Mannose-6-Phosphate	M-1-P	Mannose-1-Phosphate
GDP-Man	Guanosine Diphosphate Mannose	GDP-Fuc	Guanosine Diphosphate Fucose
UDP-Rha	Uridine Diphosphate Rhamnose	UDP-Gal	Uridine Diphosphate Galacturonate
UDP-GlcA	Uridine Diphosphate Glucuronate	UDP-Xyl	Uridine Diphosphate Xylose
UDP-Api	Uridine Diphosphate Apiose	UDP-Arap	Uridine Diphosphate Arabinose, Pyranose form
UDP-Araf	Uridine Diphosphate, Furanose form	FDA	Fructose-Bisphosphate Aldolase
GPI	Phosphoglucose Isomerase	PGM	Phosphoglucomutase
SP	Starch Phosphorylase	AGP	Adenosine Diphosphoglucose Pyrophosphorylase
SSS	Soluble Starch Synthase	GBSS	Granule-bound Starch Synthase
SBE	Starch Branching Enzyme	UGP	UDP-Glucose Pyrophosphorylase
CesA	Cellulose Synthase Catalytic Subunit	Csl	Cellulose Synthase
MPI	Phosphomannose Isomerase	PMM	Phosphomannomutase
MGP	Mannose-1-Phosphate Guanylyltransferase	GMD	GDP-Mannose 4,6-Dehydratase
INV	Invertase	cwINV	Cell Wall Invertase
SPS	Sucrose-Phosphate Synthase	SUS	Sucrose Synthase
RHM	Rhamnose Biosynthetic Enzyme	UGE	UDP-Glucose 4-Epimerase
UGD	UDP-Glc Dehydrogenase	UXS/AXS	UDP-Glucuronate Decarboxylases
UAE	UDP-Arabinose 4-Epimerase	UAM	UDP-Arabinose Mutase
ABA	Abscisic Acid	GA	Gibberellin
SA	Salicylic Acid	JA	Jasmonate
BR	Brassinolide	MVA	Mevalonic Acid
IPP	Isopentenyl Pyrophosphate	Zx	Zeaxanthin
ATV	All-trans Violaxanthin	9-*cis*-Nx	9-*cis*-Neoxanthin
GPP	Geranyl- Pyrophosphate	FPP	Farnesyl Pyrophosphate
GGPP	Geranylgeranyl Pyrophosphate	CPP	Cuban Pyrophosphate
KA	Kaurenoic Acid	Sha	Shikimic Acid
Cha	Chorismic acid	ICA	Isochorismic Acid
L-Aro	L-Arogenate	Ca	Cinnamic Acid
Ba	Benzoic Acid	ALA	Linolenic Acid
HPALA	13-Hydrogen Peroxide Linolenic Acid	EALA	12,13-Epoxylinolenic Acid
OPDA	12-Oxophytodienoic Acid	CR	Campesterol
CHOLK	Cholesten-3-Ketone	CN	Campestanol
OCN	6-Oxocampestanol	CT	Cathasterone
6DCT	6-Deoxycathasterone	TE	Teasterone
6DTE	6-Deoxyteasterone	3DT	3-Dehydrocathasterone
6D3DT	6-Deoxy-3-Dehydrocathasterone	TY	Typhasterol
6DTY	6-Deoxytyphasterol	CS	Castasterone
6DCS	6-Deoxycastasterone	OTS1	Open Stomata 1
SnRK2	Sucrose Non-fermenting 1-related Kinase	MAPK	Mitogen Activated Protein Kinase
ZEP	Zeaxanthin Epoxidase	NCED	9-*cis*-Epoxycarotenoid Dioxygenase
SDR	Short-chain Dehydrogenase/reductase	AAO	ABA Aldehyde Oxidase
ICS	Isochorismate Synthase	PAL	Phenylalanine Ammonialyase
GGPS	Geranylgeranylpyrophosphate	CPS	Copalyl Pyrophosphate Synthase
KS	Kaurene Synthase	KO	Kaurene Oxidase
GA	Gibberellin Oxidase	LOX	Lipoxygenase
AOS	Allene Oxide Synthase	AOC	Allene Oxide Cyclase
OPR	12-Oxophytodienoate Reductase	DWF	Trans-Cinnamate 4-Hydroxylase
ROT	C-23 Hydroxylase	CPD	Coumarate-3-Hydroxylase
